# Early postoperative seizures (EPS) in patients undergoing brain tumour surgery

**DOI:** 10.1038/s41598-020-70754-z

**Published:** 2020-08-13

**Authors:** Tunc Faik Ersoy, Sami Ridwan, Alexander Grote, Roland Coras, Matthias Simon

**Affiliations:** 1 Department of Neurosurgery, Evangelisches Klinikum Bethel, Kantensiek 11, 33617 Bielefeld, Germany; 2 Department of Neurosurgery, Paracelsus-Klinik Osnabrück, Am Natruper Holz 69, 49076 Osnabrück, Germany; 3 Institute of Neuropathology, University Hospital Erlangen, Schwabachanlage 6, 91054 Erlangen, Germany

**Keywords:** Epilepsy, Surgical oncology

## Abstract

Early postoperative seizures (EPS) are a common complication of brain tumour surgery. This paper investigates risk factors, management and clinical relevance of EPS. We retrospectively analysed the occurrence of EPS, clinical and laboratory parameters, imaging and histopathological findings in a cohort of 679 consecutive patients who underwent craniotomies for intracranial tumours between 2015 and 2017. EPS were observed in 34/679 cases (5.1%), with 14 suffering at least one generalized seizure. Patients with EPS had a worse postoperative Karnofsky performance index (KPI; with EPS, KPI < 70 vs. 70–100: 11/108, 10.2% vs. 23/571, 4.0%; *p* = 0.007). Preoperative seizure history was a predictor for EPS (none vs. 1 vs. ≥ 2 seizures: *p* = 0.037). Meningioma patients had the highest EPS incidence (10.1%, *p* < 0.001). Cranial imaging identified a plausible cause in most cases (78.8%). In 20.6%, EPS were associated with a persisting new neurological deficit that could not otherwise be explained. 34.6% of the EPS patients had recurrent seizures within one year. EPS require an emergency work-up. Multiple EPS and recurrent seizures are frequent, which indicates that EPS may also reflect a more chronic condition i.e. epilepsy. EPS are often associated with persisting neurological worsening.

## Introduction

Early postoperative seizures (EPS) are a common complication of brain tumour surgery. EPS are often categorized as acute symptomatic seizures^1,2^. They are usually felt to reflect acute medical or surgical conditions that may require emergency treatment. This includes haemorrhages, infectious complications and electrolyte disturbances, but also systemic infections and cardiopulmonary disorders resulting in hypotension and hypoxia. Hence, EPS may have potentially severe consequences. They may result in significant and often persisting (neurological) morbidity and reduced quality of life. Furthermore, they usually prolong the patient’s hospital stay. Potential negative consequences include a delayed transfer for rehabilitation therapy, an overall prolonged rehabilitation and, importantly, delayed adjuvant therapy. This latter aspect is of considerable importance e.g. in patients with gliomas and metastasis who will often not realize the benefits of surgery if adjuvant therapy is withhold. In addition, many patients with brain metastases require more or less urgent treatment for their systemic disease.

There is also the issue of distinguishing between incidental or acute symptomatic seizures with no or a very low risk of recurrent seizures and true postoperative chronic epilepsy^1–3^. The latter condition requires chronic treatment with antiepileptic drugs and comes with relevant socioeconomic sequelae such as restriction of driving privileges. This may be a particularly important issue for patients with benign tumours such as many meningiomas who have a good chance of a surgical cure of their tumour. In such cases, the risk of recurrent seizures may well be their only (neurological) health concern^4,5^.

There is a growing interest in tumour-associated epilepsy^6,7^. However, relatively few investigators have focused specifically on EPS^8,9^. Consequently, questions regarding the necessary diagnostic work-up and the use of antiepileptic drugs in cases with EPS are difficult to answer. For the present study, we have therefore reviewed our recent institutional experience with EPS after brain tumour surgery between 2015 and 2017.

## Materials and methods

### Patients

We identified all 679 consecutive patients who underwent a craniotomy for an intracranial tumour between January 2015 and August 2017 in the Department of Neurosurgery, Evangelisches Klinikum Bethel, Bielefeld, Germany, by searching the departmental electronic database. Patients operated for medication-refractory epilepsy within the epilepsy surgery program were excluded from this analysis, i.e. all cases specifically referred to us following a presurgical work-up aiming at the establishment of a surgical concept for epilepsy rather than tumour control^10^. During the study period, we used the current (= 2009) ILAE (International League Against Epilepsy) definition of pharmacoresistant epilepsy, i.e. “… failure of adequate trials of two tolerated, appropriately chosen and used antiepileptic drug schedules (whether as monotherapies or in combination) to achieve sustained seizure freedom”^11^.

### Clinical data

Pertinent clinical data and follow-up information were retrospectively retrieved through a chart review and entered in an electronic database. Clinical parameters included age at surgery, gender, histopathological diagnosis, surgery for tumour recurrence, extent of resection, pre- and postoperative (time of discharge) Karnofsky Performance Index (KPI), occurrence of seizures prior to operation and their frequency, type of the seizure (according to the current ILAE (International League Against Epilepsy) classification)^12^ and preoperative anticonvulsive therapy. The preoperative MRI work-up was reviewed in each case and the respective tumour location was recorded including specifically the following items: supra- vs. infratentorial and intra- vs. extra-axial growth as well as involvement or compression of the frontal, temporal, central or insular lobe. Tumours affecting the latter cerebral structures are believed to be particularly prone to cause epilepsy^13^. EPS were defined as (generalized) involuntary movements, abnormal sensory phenomena or an altered mental status that could not otherwise be explained, occurring within 30 days post-surgery, and categorized according to the current ILAE classification^8,12,14^. Acute electroencephalograms were ordered in cases in which the diagnosis of an EPS was questionable^8^. We specifically reviewed all clinical, laboratory and in particular imaging data from all patients with EPS in order to identify their likely cause.

Maximum tumour diameter and perifocal oedema > 1 cm were assessed in all meningioma patients using axial contrast-enhanced T1- and axial FLAIR-weighted scans, respectively^4^. Oedema formation and large tumour size have been associated with preoperative seizures in meningioma patients by some authors^4,15,16^. We also reviewed all available early (< 24 h) postoperative imaging studies from cases undergoing supratentorial meningioma surgery. Specifically, we recorded extra-axial (epi- or subdural) bleeds and pneumocephalus with mass effect, cerebral contusions, and measurable (i.e. > 0.5 cm) hematomas or bleeds with mass effect in the resection cavity as well as new small/perforator and territorial infarcts. Such imaging findings may cause neurological deficits and/or have clinical consequences ranging from prolonged observation to revision surgery^17^. Of note, they would also be regarded as plausible causes for an acute symptomatic seizure^2^.

### Histopathology

All neuropathological analyses were performed at the Institute of Neuropathology, University Hospital Erlangen, Germany. This included immunohistochemistry and molecular genetic analysis if required by the WHO 2016 classification or its 2007 predecessor, or whenever such additional investigations were deemed useful by the responsible neuropathologist.

### Statistical analysis

For statistical analyses, we used a commercially available software (IBM SPSS Statistics for Windows, Version 25.0, IBM Corp., Armonk, NY). Standard procedures (Fisher exact test, chi-square test, linear-by-linear association [Mantel–Haenszel test] and Student-t-test) were used for univariate analyses as indicated. Two-sided tests were employed throughout and *p* values < 0.05 were considered significant. For multivariate analyses, we used logistic regression modelling (inclusion procedure).

### Ethical approval

The study was approved by the responsible institutional research committee and all procedures were in accordance with its ethical standards and with the 1964 Helsinki declaration and its later amendments (Ethikkommission der Ärztekammer Westfalen-Lippe und der Westfälischen Wilhelms-Universität Münster, Germany, Az 2018-484-f-S).

### Informed consent

The responsible institutional research committee and local law do not require informed consent for this study.

## Results

### Patient cohort

We studied a total of 679 surgical cases, i.e. 679 procedures performed in 630 patients. Median age was 61.0 years. The series comprises more females (379, 60.2%) than males (251, 39.8%). The median pre- and postoperative KPI was both 90. 103 (15.2%) patients had surgery for a recurrent tumour. 137 (20.2%) surgeries were performed for infratentorial and 542 (79.8%) surgeries for supratentorial tumours. The latter figure includes 4/16 tentorial meningiomas with at least some extension into the supratentorial compartment. Twenty-six cases had open microsurgical biopsies (3.8%). The most frequent histology was meningioma (all WHO grades, N = 218, 32.1%), followed by glioblastoma (N = 177, 26.1%), and metastasis (N = 138, 20.5%). The histopathological diagnoses are detailed in Table 1.

**Table 1:** Histopathological diagnoses.

Histology^b^	N	%
Meningioma WHO grade I	168	24.7
Atypical meningioma WHO grade II	47	6.9
Anaplastic meningioma WHO grade III	3	0.4
Hemangiopericytoma/ SFT WHO grades II & III	3	0.4
(Vestibular) schwannoma	18	2.7
Glioblastoma multiforme, IDH wildtype, WHO grade IV	159	23.4
Glioblastoma multiforme, IDH mutated, WHO grade IV	7	1.0
Giant cell glioblastoma WHO grade IV	6	0.9
Gliosarcoma WHO grade IV	5	0.7
Anaplastic astrocytoma, IDH mutated, WHO grade III	12	1.8
Anaplastic astrocytoma, IDH wildtype, WHO grade III	5	0.7
Astrocytoma, IDH mutated, WHO grade II	10	1.5
(Anaplastic) astrocytoma, NOS, WHO grades II & III	2	0.3
Anaplastic oligodendroglioma, IDH mutated, WHO grade III	12	1.8
Oligodendroglioma, IDH mutated, WHO grade II	7	1.0
Glioneuronal tumours	8	1.2
Pilocytic astrocytoma	7	1.0
Pleomorphic xanthoastrocytoma WHO grade II	1	0.1
Ependymoma WHO grades II & III	12	1.8
Subependymoma WHO grade I	2	0.3
Medulloblastoma & PNET	3	0.4
Craniopharyngioma	5	0.7
Pituitary adenoma	6	0.9
Hemangioblastoma WHO grade I	10	1.5
Lymphoma	17	2.5
Metastasis	139	20.5
Other	5	0.7
Total	679	100.0^a^

^a^Due to round-off error, the percentages add up to < 100%.

^b^WHO, World Health Organization; SFT, solitary fibrous tumour; IDH, isocitrate dehydrogenase 1 and 2; NOS, not otherwise specified (= no molecular genetic studies performed); PNET, primitive neuroectodermal tumour.

In total, 155 patients (22.8%) presented with seizures prior to surgery, with approximately half of them (82, 12.1%) reporting a history of multiple (≥ 2) seizures. Preoperative generalized seizures occurred in 83 cases (12.2%). Antiepileptic treatment was prescribed in 141 of the 155 cases (93.0%) with preoperative seizures (levetiracetam: 121; other monotherapy: 6; combination therapy including levetiracetam: 13; other combination therapy: 1). The remaining 14 cases were not felt to have presented with seizures by their referring physicians and the treating neurosurgeons at the time of their surgery. Patients without a seizure history did not receive (prophylactic) anticonvulsive treatment. A postoperative steroid taper starting with 16–24 mg dexamethasone on day 1 was routinely prescribed in cases with significant brain oedema and mass effect.

### Patients with early postoperative seizures

EPS seizures were observed in 34/679 cases (5.1%), with 17 patients (2.5%) suffering multiple seizures. 14 patients (2.1%) had at least one generalized seizure. More than half of these patients had seizures within the first three days following surgery and 29/34 (85.3%) within the first 7 days. All cases suffering an EPS are described in detail in Table 2.

**Table 2:** Demographic and clinical data of the 34 patients with EPS.

No	Sex^a^/age	Histology^b^	Location^c^	Preoperative seizures^d^	EPS^d,e^	Seizure cause^f^	Surgical revision	KPI^g^ (preop./discharge)	Seizure-related deficits and complications	Seizure w/in 1 year^j^
1	f/74	Meningioma °I	R medial sphenoidal wing	No	D6: Focal cognitive (aphasia) sz	?	No	100/100	Temporary aphasia & confusion	N/A
2	f/60	Meningioma °I	R temporooccipital convexity	No	D3: Focal aware motor szs	ICB	No	90/90	No	Yes
3	f/74	Meningioma °I	L frontal falx	No	D1: Gen tonic–clonic sz	?	No	90/90	No	Yes
4	f/68	Meningioma °I	R frontal convexity	No	D5: Unknown onset tonic–clonic sz	ICB	No	20/70	No	No
5	f/42	Meningioma °I	L > R olfactory groove	No	D6: Gen tonic–clonic sz	?	No	100/90	No	No
6	f/61	Meningioma °I	R medial sphenoidal wing	No	D1: Focal aware motor szs	Oedema	No	50/30	Persisting stupor	No
7	m/43	Meningioma °I	L frontal convexity	No	D1: Unknown onset tonic–clonic + focal aware motor szs	Infarction	No	100/100	No	Yes
8	f/69	Meningioma °I	L frontal convexity	No	D1: Focal aware motor szs	SDH	Yes	90/70	Temporary aphasia & confusion	No
9	f/71	Meningioma °I	Planum sphenoidale (+ l petrosal)	No	D0: Focal aware motor szs	Oedema, ICB	No	90/70	No	No
10	f/71	Meningioma °I (2x)	L frontal convexity	Focal cognitive (aphasia) sz	D4: Focal cognitive (aphasia) sz	EDH	Yes	90/80	No	N/A
11	f/41	Meningioma °I	R temporal convexity (multiple)	Focal impaired awareness szs	D6: Focal aware motor szs	Multiple tumours	No	40/40	No	No
12	f/77	Meningioma °I	L frontal convexity	Focal aware motor szs	D1: Gen tonic–clonic szs (progressing to status epilepticus)	ICB	No	90/0^ h^	No	N/A
13	f/66	Meningioma °I (recurrent)	R central parasagittal	No	D1: Focal aware motor sz	Oedema	No	100/90	Temporary hemiparesis, persisting foot drop	No
14	f/43	Meningioma °I (recurrent)	R fronto-basal	No	D2: Gen tonic–clonic sz	ICB	No	90/90	No	No
15	f/70	Atyp. Meningioma °II	L > R central falx	No	D2: Focal aware motor szs	ICB	No	100/100	No	No
16	f/78	Atyp. Meningioma °II	L frontal falx	No	D1: Focal aware motor szs (progressing to tonic–clonic)	ICB	No	100/50	Temporary hemiparesis, persisting aphasia and confusion	No
17	f/6 k	Atyp. Meningioma °II	L frontal falx	No	D7: Unknown onset motor sz	Oedema	No	100/100	No	No
18	m/81	Atyp. Meningioma °II	L frontotemporal convexity	Focal impaired awareness szs	D1: Focal aware motor szs	Infarction, SDH	No	60/0^i^	Pneumonia	No
19	f/75	Atyp. Meningioma °II	L > R frontal convexity (multiple)	Focal aware motor szs	D8: Focal aware motor szs	Multiple tumours	No	80/80	No	Yes
20	f/81	Atyp. Meningioma °II	R lateral sphenoid wing	Focal aware motor szs	D5: Gen tonic–clonic sz	Oedema	No	20/50	No	No
21	f/37	Atyp. Meningioma °II	L frontal convexity	Gen tonic–clonic sz	D2: Unknown onset motor szs	Oedema, hypernatremia	No	20/40	No	Yes
22	m/69	Atyp. Meningioma, °II (recurrent)	R > L parieto-occipital parasagittal	Focal aware sensory szs (progressing to bilat tonic–clonic)	D10: Gen atonic szs	?	No	90/60	Persisting confusion	Yes
23	m/36	Astrocytoma °II, IDH mt, no 1p/19q del	L temporal	No	D2: Focal cognitive (aphasia) sz	Awake craniotomy	No	100/100	Temporary aphasia	Yes
24	m/23	Astrocytoma °II, IDH mt, no 1p/19q del	L temporoinsular	Focal aware autonomic szs (progressing to bilat tonic–clonic)	D8: Focal aware motor + cognitive (aphasia) szs	SDH, awake craniotomy	No	90/80	Temporary hemiparesis and confusion, persisting aphasia	Yes
25	m/66	sGBM, IDH mt	R temporal	Focal impaired awareness szs	D5: Focal impared awareness + cognitive (aphasia) szs	EDH, ICB	No	90/90	Temporary dysarthria & confusion	No
26	m/70	sGBM, IDH mt	R frontal	No	D3: Gen tonic–clonic sz	EDH, ICB	Yes	80/80	Temporary confusion	No
27	m/76	GBM, IDH wt	R frontal	No	D8: Focal aware motor + cognitive (aphasia) sz	ICB	No	70/40	Persisting aphasia & hemiparesis	N/A
28	m/60	GBM, IDH wt	L temporal	No	D3: Focal aware motor + cognitive (aphasia) szs	SDH, ICB, hyponatremia	Yes	100/60	Temporary hemiparesis & confusion, persisting aphasia	Yes
29	m/42	GBM, IDH wt	R frontal	No	D7: Gen tonic–clonic sz	Meningitis	No	70/70	No	No
30	m/84	GBM, IDH wt	R postcentral	No	D7: Unclassified sz	ICB	No	90/90	Temporary aphasia & confusion	N/A
31	m/57	GBM, IDH wt	R frontal cingulum	Focal aware motor sz	D0: Focal aware motor szs	Infarction	No	100/100	No	N/A
32	m/54	Metastasis	R central (+ l cerebellar)	No	D3: Gen tonic–clonic sz	EDH	No	90/60	No	N/A
33	f/50	Metastasis (recurrent)	L temporomesial	No	D4: Gen tonic–clonic sz	Meningitis	No	90/80	No	N/A
34	m/29	Vestibular schwannoma	R cerebellopontine angle	Gen tonic–clonic sz	D13: Gen tonic–clonic sz	Hydrocephalus, EVD	No	80/70	No	No

^a^f, female; m, male.

^b^Atyp., atypical; °, WHO grade; GBM, glioblastoma multiforme; sGBM, secondary glioblastoma; wt, wildtype; mt, mutated; del, deleted.

^c^L, left; R, right; multiple, ≥ 2.

^d^Gen, generalized; sz/szs; seizure/seizures.

^e^D, postoperative day of the (first) EPS.

^f^ ICB, intracerebral bleeding; SDH, subdural hematoma; EDH, epidural hematoma; EVD, external ventricular drain; ?, unknown.

^g^KPI, Karnofsky performance index.

^h^Large cardiogenic MCA infarction, death due to sepsis.

^i^Pneumonia, death due to respiratory insufficiency.

^j^w/in, within; N/A, not available.

We found no correlations between age, sex or preoperative KPI with the occurrence of EPS in the overall cohort (Table 3). Patients with EPS had a worse postoperative KPI (patients with EPS, KPI < 70 vs. 70–100: 11/108, 10.2% vs. 23/571, 4.0%; *p* = 0.007). Preoperative seizure history was a risk factor for EPS. EPS were seen in 22/524 (4.2%) cases with no preoperative seizure, but in 4/73 (5.5%) patients with a single and 8/82 (9.8%) cases with multiple preoperative seizures (none vs. 1 vs. ≥ 2 seizures: *p* = 0.037; none vs. any preoperative seizure: *p* = ns; 0–1 vs. ≥ 2 seizures: *p* = 0.035; Table 3). EPS were observed in 10/141 (7.1%) patients on anticonvulsive medication and in 2/14 (14.3%) cases who had preoperative seizures, yet no anticonvulsive treatment.

**Table 3:** Risk factors for EPS in N = 679 brain tumour operations.

Variable^a^	Mean ± SD/ N^b^	EPS	No EPS	*p*^c^
Age (years)	59.8 ± 15.0	60.6 ± 16.4	59.8 ± 14.9	ns
**Sex**				
Female	404	20/404 (5.0%)	384/404 (95.0%)	ns
Male	275	14/275 (5.1%)	261/275 (94.9%)	
**Preoperative KPI**				
KPI < 70	69	6/69 (10.2%)	63/69 (91.3%)	ns
KPI 70–100	610	28/610 (4.6%)	582/610 (95.4%)	
Postoperative KPI				
KPI < 70	108	11/108 (10.2%)	97/108 (89.8%)	0.007*
KPI 70–100	571	23/571 (4.0%)	548/571 (96.0%)	
**Preoperative seizures**				
No seizure	524	22/524 (4.2%)	502/524 (95.6%)	ns
≥ 1 seizure	155	12/155 (7.7%)	143/155 (92.3%)	
No or 1 seizure	597	26/597 (4.4%)	571/597 (95.6%)	0.035*
≥ 2 seizures	82	8/82 (9.8%)	74/82 (90.2%)	
Anticonvulsive	141	10/141 (7.1%)	131/141 (92.9%)	ns
No anticonvulsive	537	24/537 (4.5%)	513/537 (95.5%)	
**Histology**				
Glioblastoma	177	7/177 (4.0%)	170/177 (96.0%)	< 0.001*
Other glioma	64	2/64 (3.1%)	62/64 (96.9%)	
Meningioma	218	22/218 (10.1%)	198/218 (89.9%)	
Metastasis	139	2/139 (1.4%)	137/139 (98.6%)	
Other	81	1/81 (1.2%)	80/81 (98.8%)	
**Location**				
Supratentorial	542	33/542 (6.1%)	509/542 (93.9%)	0.007*
Infratentorial	137	1/137 (0.7%)	136/137 (99.3%)	
Intra-axial	406	11/406 (2.7%)	395/406 (97.3%)	0.001*
Extra-axial	273	23/273 (8.4%)	250/273 (91.6%)	
Frontal, central, temporal and/or insular lobe involvement/compression	452	32/452 (7.1%)	420/452 (92.9%)	< 0.001*
No	227	2/227 (0.9%)	225/227 (99.1%)	
**Surgical procedure**				
Resection	653	34/653 (5.2%)	619/653 (94.8%)	ns
Biopsy	26	0/26 (0%)	26/26 (100%)	
Surgery for recurrence	103	4/103 (3.9%)	99/7,103 (96.1%)	ns
First tumours surgery	576	30/576 (5.2%)	546/576 (94.8%)	

^a^KPI, Karnofsky performance index.

^b^Age: Mean ± SD, standard deviation; all other variables: N, number of surgical cases.

^c^ns, not significant.

*p < 0.05.

Only 1/137 (0.7%) patient with an infratentorial tumour, but 33/542 (6.1%, *p* = 0.007) patients with supratentorial growths suffered an EPS. EPS were seen significantly more often following surgery for tumours involving or compressing the frontal, central, temporal and/or insular lobes (32/452, 7.1% vs. 2/227, 0.9%, *p* < 0.001). At least some involvement of the primary sensorimotor cortex was seen in 83 patients, 4 of which experienced an EPS (4.8%). Extra-axial growths carried an increased risk for EPS (extra-axial vs. intra-axial: 23/273, 8.4% vs. 11/406, 2.7%, *p* = 0.001). More precisely, the risk of EPS varied significantly with tumour histology. The majority of the patients suffering an EPS underwent surgery for a meningioma (22/34, 64.7%). EPS were seen in 22/218 (10.1%) cases with meningioma, but only in 7/177 (4.0%) with glioblastoma, 2/64 (3.1%) with other gliomas (i.e. diffuse astrocytoma or oligodendroglioma WHO grades II and III, pilocytic astrocytoma, pleomorphic xanthoastrocytoma and glioneuronal tumours), 2/139 (1.4%) with metastasis, and 1/81 (1.2%) with other histologies (*p* = 0.001). Repeat surgery was not associated with an increased risk for EPS (Table 3).

Next, we performed a multivariate binary logistic regression analysis. Using preoperative seizures (none, single, ≥ 2), tumour location (infra- vs. supratentorial growth and involvement/ compression of the frontal, central, temporal and/or insular lobes vs. not) and histology (meningioma, glioblastoma, other glioma vs. metastases and all “other” histologies combined) as covariates, the analysis revealed only histology (*p* = 0.007) as an independent predictor of EPS. Specifically, patients with meningiomas had a 4.79-fold (95% CI 1.37–16.78, *p* = 0.014) increased relative risk for suffering EPS using the combined metastases and “other” histologies subgroup as reference category.

### Early postoperative seizures in meningioma patients

The majority of our EPS cases had surgery for a meningioma. Preoperative seizure history, supratentorial tumour location, growth in association with the frontal, central, temporal and/or insular lobes and a worse postoperative KPI were associated with EPS not only in the overall cohort but also in the meningioma patient subgroup. Likely due to the limited sample size, only the latter two correlations proved statistically significant (Table 4). We found no correlation between oedema and EPS, while patients with EPS had indeed somewhat larger tumours (largest tumour diameter, axial T1-weighted contrast-enhanced images, EPS vs. no EPS: 471.4 ± 243.3 vs. 391.8 ± 184.2 mm, *p* = ns; Table 4).

**Table 4:** Risk factors for EPS in N = 218 meningioma operations.

Variable^a^	Mean ± SD/ N^b^	EPS	No EPS	p^c^
Age (years)	61.3 ± 14.3	64.3 ± 14.0	61.0 ± 14.3	ns
**Sex**				
Female	169	19/169 (11.2%)	150/169 (88.8%)	ns
Male	49	3/49 (6.1%)	46/49 (93.9%)	
**Preoperative KPI**				
KPI < 70	21	6/21 (28.6%)	15/21 (71.4%)	ns
KPI 70–100	197	16/197 (8.1%)	181/197 (91.9%)	
**Postoperative KPI**				
KPI < 70	40	9/40 (22.5%)	31/40 (77.5%)	0.004*
KPI 70–100	178	13/178 (7.3%)	165/178 (92.7%)	
**Preoperative seizures**				
No seizure	163	14/163 (8.6%)	149/163 (91.4%)	ns
≥ 1 seizure	55	8/55 (14.5%)	47/55 (85.5%)	
No or 1 seizure	188	16/188 (8.5%)	172/188 (91.5%)	ns
≥ 2 seizures	30	6/30 (20.0%)	24/30 (80.0%)	
Anticonvulsive	46	6/46 (13.0%)	40/46 (87.0%)	ns
No anticonvulsive	172	16/172 (9.3%)	156/172 (90.7%)	
**Location**				
Supratentorial	186	22/186 (11.8%)	164/186 (88.2%)	ns
Infratentorial	32	0/32 (0%)	32/32 (100%)	
Convexity/falx	125	16/125 (12.8%)	109/125 (87.2%)	ns
Supratentorial skull-base	56	5/56 (8.9%)	51/56 (91.1%)	
Other	37	1/37 (2.7%)	36/37 (97. 3%)	
Frontal, central, temporal and/or insular lobe compression	166	21/166 (12.7%)	145/166 (87.3%)	0.031*
No	52	1/52 (1.9%)	51/52 (98.1%)	
**Surgical procedure**				
Resection	217	22/217 (10.1%)	195/217 (89.9%)	ns
Biopsy	1	0/1 (0%)	1/1 (100%)	
Complete resection	194	21/194 (10.8%)	173/194 (88.2%)	ns
STR or biopsy	24	1/24 (4.2%)	23/24 (95.8%)	
Surgery for recurrence	30	3/30 (10.0%)	27/30 (90.0%)	ns
First tumour surgery	188	19/188 (10.1%)	169/188 (89.9%)	
**Histology**				
WHO grade I	168	14/168 (8.3%)	154/168 (91.7%)	ns
WHO grade II	47	8/47 (17.0%)	39/47 (83.0%)	
WHO grade III	3	0/3 (0%)	3/3 (0%)	
Tumour size (mm)	400.0 ± 191.9	471.4 ± 243.3	391.8 ± 184.2	ns
**Postoperative imaging**^**d**^				
Extra-axial hematoma	19	3/19 (15.8%)	16/19 (84.2%)	ns
No	147	19/147 (12.9%)	128/147 (87.1%)	
Resection cavity hematoma	67	13/67 (19.4%)	54/67 (80.6%)	ns**
No	99	9/99 (9.1%)	90/99 (90.9%)	
Brain contusion	67	8/67 (11.9%)	59/67 (88.1%)	ns
No	99	14/99 (14.1%)	85/99 (85.9%)	
Infarct	31	4/31 (12.9%)	27/31 (87.1%)	ns
No	135	18/135 (13.3%)	117/135 (86.7%)	
Pneumocephalus	24	2/24 (8.3%)	22/24 (91.7%)	ns
No	142	20/142 (14.1%)	122/142 (85.9%)	

^a^KPI, Karnofsky performance index; complete resection, Simpson grade 1–3; STR/biopsy, Simpson grade 4–5.

^b^Age & tumour size: Mean ± SD, standard deviation; all other variables: N, number of surgical cases.

^c^ns, not significant.

^d^supratentorial tumours only.

*p < 0.05.

**p = 0.055.

All meningioma cases with EPS had supratentorial surgery. Early postoperative imaging studies could be made available in 166/186 (89.2%) patients (151 cCT, 15 MRI) with supratentorial meningiomas. Neither extra-axial hematomas, nor brain contusions, postoperative infarcts, resection cavity hematomas or pneumocephalus were found to significantly predict EPS. However, there was a statistical trend for an association between EPS and resection cavity hematoma (*p* = 0.055).

### Early postoperative seizures: work-up, clinical relevance and outcome

**Figure 1:**
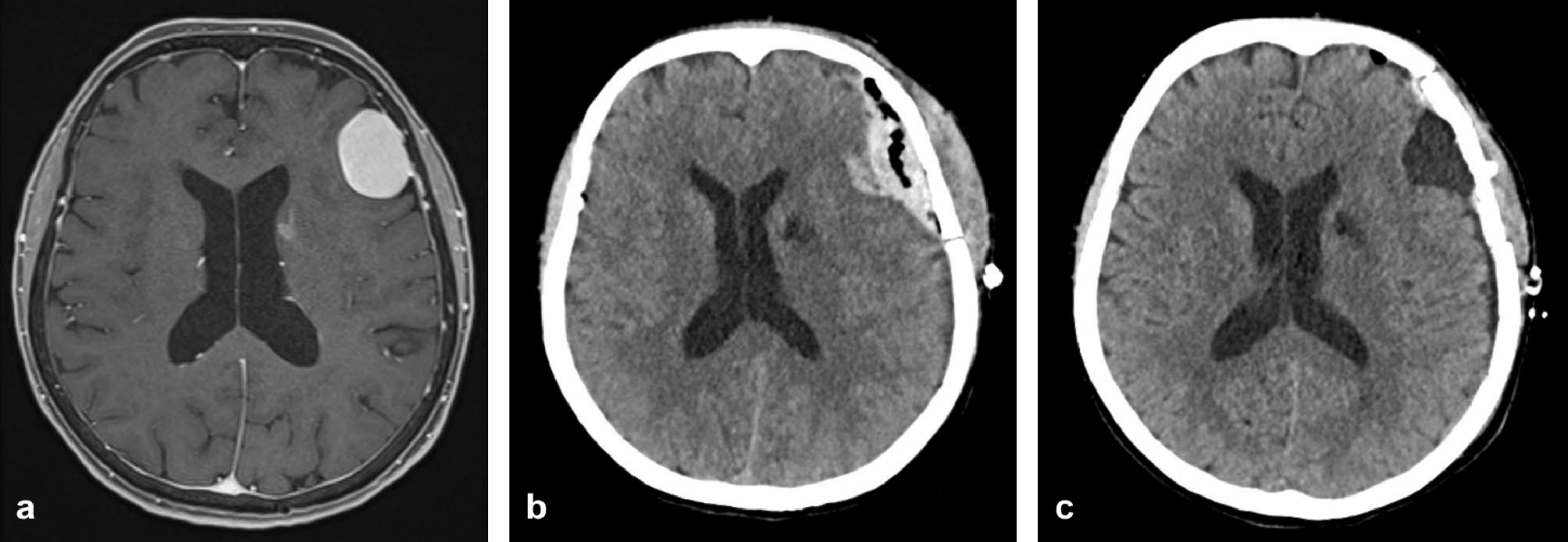
(**a**) A 71-year old female patient (no. 10) with a history of one (focal) preoperative seizure, who underwent surgery for a left frontal convexity meningioma. (**b**) On the fourth postoperative day, the patient suffered another focal seizure, yet exhibited no focal or other neurological deficit. The emergency CT scan showed a relevant epidural hematoma, which was surgically removed. (**c**) Postoperative CT scan after epidural clot removal. The patient’s further clinical course was uneventful.

Following an EPS, all except one of the 34 patients received cranial imaging within 24 h. MR and/or CT scans revealed a plausible cause for the seizure in 26/33 (78.8%) of the cases (Table 2). This includes 22 patients managed conservatively and four patients who required surgery for removal of epidural, subdural and/or bleeds into the resection cavity. Of note, one of these cases reported a preoperative seizure history, yet no postoperative or postictal deficit (Fig. 1). However, the size of the (epidural) bleed was felt to warrant surgery. Only two patients undergoing revision surgery recovered to a KPI at discharge of 80 or better (Table 2). The one case (no. 34) without emergency CT or MR scanning presented initially with a generalized seizure. His postoperative MRI was unremarkable. He suffered a second seizure with identical semiology several days later, from which he recovered quickly and completely. A CT scan was obtained a few days later showing no acute pathology.

Laboratory testing diagnosed relevant serum electrolyte disturbances in two cases (1 hyponatremia: Na^+^  = 126 mmol/l, 1 hypernatremia: Na^+^  = 159 mmol/l; Table 2). Meningitis was believed to be responsible for seizures in two cases. These two cases made a good recovery following appropriate antibiotic treatment (Table 2). Awake craniotomies and intraoperative cortical electrostimulation were thought to play a causative role in two other cases, and a temporary external ventricular drain in one patient.

Medical management of EPS patients relied heavily on levetiracetam. 20/22 (90.9%) of cases with de-novo EPS were treated with levetiracetam. In the remaining two cases, no anticonvulsive treatment was initiated. Ten EPS patients with preoperative seizures had their levetiracetam dose increased or adjusted based on serum level determinations. In two cases with preoperative epilepsy and multiple EPS, who were already on levetiracetam, concomitant medication with lacosamide was initiated.

EPS were associated with very relevant morbidities and even mortality. Thirteen patients (38.2%) displayed a new neurological deficit following EPS not explained by imaging or other findings, with seven of those persisting at discharge (20.6%). One patient developed pneumonia subsequent to multiple seizures and ultimately a fatal respiratory insufficiency.

Seizure recurrence after EPS was frequent. As pointed out above, 17/34 (50.0%) patients experienced multiple seizures, i.e. suffered already a recurrent seizure within 30 days of the index surgery. Patients were routinely managed by outside neurologists after discharge. Nevertheless, seizure follow-up for at least one year could be made available for 26 of these cases. Nine patients (34.6%) reported at least one other seizure within a year. Interestingly, five of these nine patients (55.6%) had a negative seizure history prior to their surgery.

## Discussion

The rate of early postoperative (= perioperative or in-hospital) seizures is usually reported as < 5–10% in most studies with some authors detailing EPS rates only for seizure-naïve patients^4,8,9,15,18,19^. We observed a 5.1% overall and 6.1% rate in patients with supratentorial tumours. Posterior fossa operations were included in our analysis primarily in order to avoid selection bias. Seizures in patients with infratentorial tumours are generally rare^19^. However, some patients with posterior fossa tumours require (temporary) ventricular drains, i.e. an (albeit minor) supratentorial operation, and certain perioperative complications thought to underlie EPS such as meningitis occur after both infratentorial and supratentorial surgeries. Interestingly, our series includes a patient undergoing surgery for a large vestibular schwannoma who required temporary CSF drainage and suffered a generalized seizure on postoperative day 13.

EPS are not benign. In a substantial number of cases, they are associated with and reflect major complications such as bleeds requiring operative revision (4/34, 11.8%) or meningitis (2/34, 5.9%). Also, early postoperative seizures per se can result in neurological worsening (13/34, 38.2%) which often persists (7/34, 20.6%). Seizures can result in pulmonary complications due to acute or silent aspirations in confused or stuporous patients. One of our cases ultimately succumbed to a pulmonary sepsis initially triggered by an early postoperative seizure.

Risk factors for EPS in our cohort included a positive preoperative seizure history, a supratentorial tumour location, tumour growth involving or compressing particularly epileptogenic brain tissues (i.e. the frontal, central, temporal and insular lobes)^13^ and, importantly, tumour histology. Meningioma patients accounted for the majority of cases and the highest overall rate (10.1%). However, contradictory results, i.e. an association with glioma histology and infiltrating growth and relatively lower perioperative seizure rates in meningioma patients have also been reported^8^. Awake craniotomy (i.e. intraoperative cortical electrostimulation) figured prominently as a risk factor for perioperative seizures in a recent study by Oushy and co-workers and was therefore felt to contribute to seizure formation in two of our cases^8^.

In accordance with the literature, we found an (albeit not statistically significantly) increased risk for EPS in patients with WHO grade II/III meningiomas, a convexity/parasagittal tumour location, perifocal oedema > 1 cm and an association with a worse postoperative KPI^4,15^. Meningioma-associated epilepsy has recently attracted some attention in the neurosurgical community. A non-skull base location, tumour size, peritumoral oedema, malignancy, tumour progression and recurrence, age and sex (as a possible corollary of a higher WHO grade), seizure history and EEG findings, clinical symptoms and surgical complications have all been (variably) associated with the risk of preoperative and postoperative seizures in meningioma patients^1,4,5,15,16,20^. We also investigated if early postoperative neuroimaging following supratentorial meningioma surgery might predict EPS. We were unable to identify significant correlations, however, there was a statistical trend for an association between EPS and the presence of a hematoma in the resection cavity.

Are EPS simply acute symptomatic seizures? By definition, acute symptomatic seizures occur within 7 days of the underlying brain insult^2^. Indeed, the majority of our cases reported seizures within the first three days following surgery and 29/34 (85.3%) within the first 7 days. Some authors have reported statistical correlations between surgical complications (including new neurological deficits) and the occurrence of early postoperative seizures^1,4^. We identified surgical complications such as haemorrhages and increased oedema, meningitis, and electrolyte disorders as the most likely cause of the seizure in 27/34 (79.4%) of cases. Clinical worsening (a possible corollary of a structural postoperative or other e.g. infectious complication) also correlated with the EPS rate in our series.

On the other hand, EPS tend to recur. 17/34 (50.0%) of our cases with EPS had a recurrent seizure within 30 days of the surgery (i.e. multiple EPS). The one year-recurrence rate was 34.6%, i.e. in a sizable proportion of EPS patients the first seizure is simply the first manifestation of epilepsy. High recurrence rates have also been published by Wirsching and co-workers (29/46, 63.0%)^1^ and Chen et al. (13/36, 36.1%)^4^. Also, the risk of EPS varies with the preoperative seizure history in this as well as in several other published cohorts^1,4^. These data suggest that not only acute perioperative brain insults but also more chronic factors such as the anatomic and metabolic alterations caused by the brain tumour contribute to early postoperative epileptogenesis.

In our view, these figures together with the adverse clinical course seen in several cases with multiple perioperative seizures justifies institution of anticonvulsive treatment already following a single perioperative seizure. Of note, we have no longer term follow-up from our patients and while we use antiepileptics quite liberally during the early postoperative period, we strongly urge patients and their physicians to re-evaluate this medication at the latest after one year. Antiepileptic medication may occasionally carry significant adverse effects. Side effects of levetiracetam, which is the current drug of choice for most neuro-oncological patients requiring anticonvulsive treatment, include e.g. fatigue, insomnia, mood and behaviour changes, headaches and decreased white blood counts^21,22^. These effects of have to be weighed against the risk of recurrent seizures with its attendant socioeconomic sequelae (e.g. restriction of driving privileges).

Given the rather high rates of adverse outcomes and recurrence after EPS, it is tempting to speculate about a role for prophylactic anticonvulsive medication. This is a controversial topic. Most believe that routine use of prophylactic anticonvulsants does not lower the risk of postoperative seizures in patients without preoperative seizures^14,23–25^. However, there are some data including a small randomized prospective trial suggesting a benefit from prophylactic levetiracetam^9^. Based on our experience and the data just outlined, we consider prophylactic levetiracetam in vulnerable patients with a high risk of EPS, e.g. an elderly patient with a large convexity meningioma.

Our study allows some conclusions with respect to the proper management of EPS. Establishing a specific diagnostic algorithm for such emergencies is important. Neuroimaging revealed the presumed cause of the seizure in the great majority (26/33, 78.8%) of our cases. In four patients, the imaging finding prompted an operative revision. We therefore feel that obtaining a CT or MRI scan after an EPS is mandatory. Meningitis and electrolyte disorders may also contribute to the formation of perioperative seizures, i.e. laboratory blood testing and a very low threshold for a lumbar puncture should be part of the diagnostic work-up.

The major limitation of our study is its retrospective design. Not all seizures reported by the patients or observed by the attending staff may have been properly documented. In addition, it is quite possible that subclinical seizures have simply escaped detection. Another shortcoming is that this is a single institutional series which somewhat limits the generalizability of our findings. Nevertheless, we present a sizable, unselected and recent experience with the diagnosis and management of EPS.

## Conclusion

Early postoperative seizures (EPS) following brain tumour surgery are common. EPS often reflect serious complications of brain tumour surgery and are associated with a relatively high rate of adverse neurological and medical sequelae. We found that structural causes not infrequently requiring surgical revision are a common cause, which suggests CT (or MR) imaging as a mandatory part of the work-up of an EPS. Multiple EPS and recurrent seizures during follow-up are frequent, which indicates that EPS are not just acute symptomatic seizures, but not uncommonly reflect a more chronic condition i.e. epilepsy. Finally, our data indicate that meningioma surgeries may carry a particularly high risk for EPS, which raises the question of an antiepileptogenic prophylaxis in selected cases.

## Data Availability

The datasets generated during and/or analysed during the current study are available from the corresponding author on reasonable request.
